# Microstructure and Wear of W-Particle-Reinforced Al Alloys Prepared by Laser Melt Injection

**DOI:** 10.3390/mi13050699

**Published:** 2022-04-29

**Authors:** Zhidong Xu, Dengzhi Wang, Wenji Song, Congwen Tang, Pengfei Sun, Jiaxing Yang, Qianwu Hu, Xiaoyan Zeng

**Affiliations:** Wuhan National Laboratory for Optoelectronics, Huazhong University of Science and Technology, Wuhan 430074, China; m202073063@hust.edu.cn (Z.X.); wjsong@hust.edu.cn (W.S.); m201972902@hust.edu.cn (C.T.); d202181055@hust.edu.cn (P.S.); m202073085@hust.edu.cn (J.Y.); huqw@hust.edu.cn (Q.H.); xyzeng@hust.edu.cn (X.Z.)

**Keywords:** laser melting injection, W particles, Al alloys, wear

## Abstract

W-particle-reinforced Al alloys were prepared on a 7075 aluminum alloy surface via laser melt injection to improve their wear resistance, and the microstructure, microhardness, and wear resistance of the W/Al layers were studied. Scanning electron microscopy (SEM) results confirmed that a W/Al laser melting layer of about 1.5 mm thickness contained W particles, and Al_4_W was formed on the surface of the Al alloys. Due to the reinforcement of the W particles and good bonding of the W and Al matrix, the melting layer showed excellent wear resistance compared to that of Al alloys.

## 1. Introduction

Aluminum alloys are widely used in automobiles, ships, aerospace, and other fields because of their low density, high specific strength, and good corrosion resistance. However, low hardness and poor wear resistance restrict their application in various fields [1,2]. Preparing particle-reinforced metal matrix composite coatings on aluminum alloys via surface engineering techniques is an effective way to improve their surface properties [3]. Laser technology has been a research hotspot in recent years [4,5]. Ayers et al., first proposed laser melt injection (LMI), which injects the additive particles into the laser melt pool directly, and then particle-reinforced metal matrix composite coatings can be formed on various metal substrate surfaces [6]. Compared with laser cladding, LMI has the advantages of low particle solubility, high surface performance, and low cracking tendency [7]. Ayers et al., prepared TiC- and WC-reinforced metal matrix composite layers on aluminum alloy substrates, and the wear resistance of the aluminum alloys was improved [8,9,10,11,12]. Vreeling et al., prepared SiC/Al composite layers on Al substrates via LMI, and found that preheating the Al substrate is an effective means of injection of SiC into the Al melt [13]. Wang et al., modified Al substrate surfaces via LMI using CeO_2_ particles, and the microstructure of the surface was suitably modified in terms of corrosion resistance [14].

In the existing literature, the most commonly used injection particles are ceramics, such as WC, SiC, TiC, etc. Ceramics are well known for their high hardness and good wear resistance, but low room-temperature toughness. Compared with ceramics such as WC, SiC, TiC, etc., W has better room-temperature toughness, and W/Al have better interface compatibility and smaller thermal and physical differences, making W an ideal reinforcing particle for aluminum alloys. Over the past few years, high-performance W/Al composite layers have been prepared via stirring friction, laser metal deposition, and laser alloying [15,16,17]. However, no studies on the laser melt injection of W-particle-reinforced metal matrix composite layers have been reported to date.

In this study, a W-particle-reinforced aluminum matrix composite layer was prepared via LMI, and the microstructure and wear behavior of the composite layer were studied.

## 2. Materials and Methods

**Figure 1:**
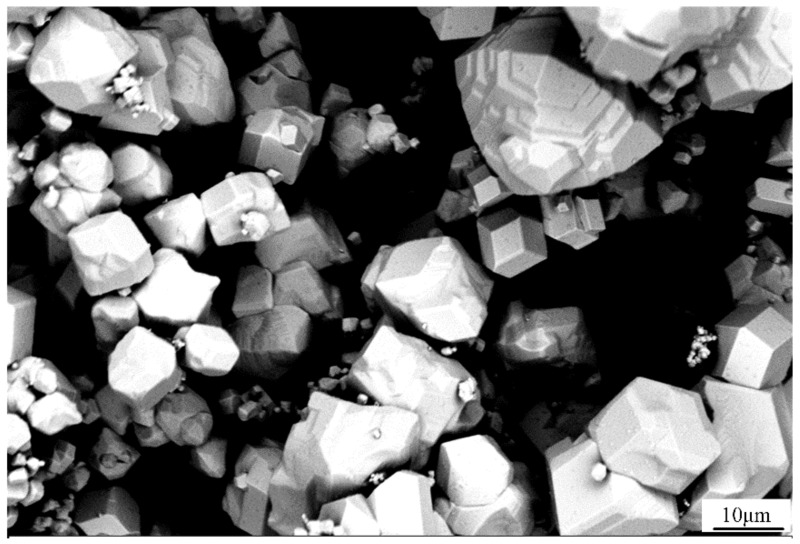
SEM of the W particles.

Tungsten (W) particles with diameter of 5–25 µm were selected as the injection particles (Figure 1), and a 7075 aluminum alloy block with dimensions of 200 mm × 150 mm × 50 mm was used as the substrate, the chemical composition of which is shown in Table 1.

**Figure 2:**
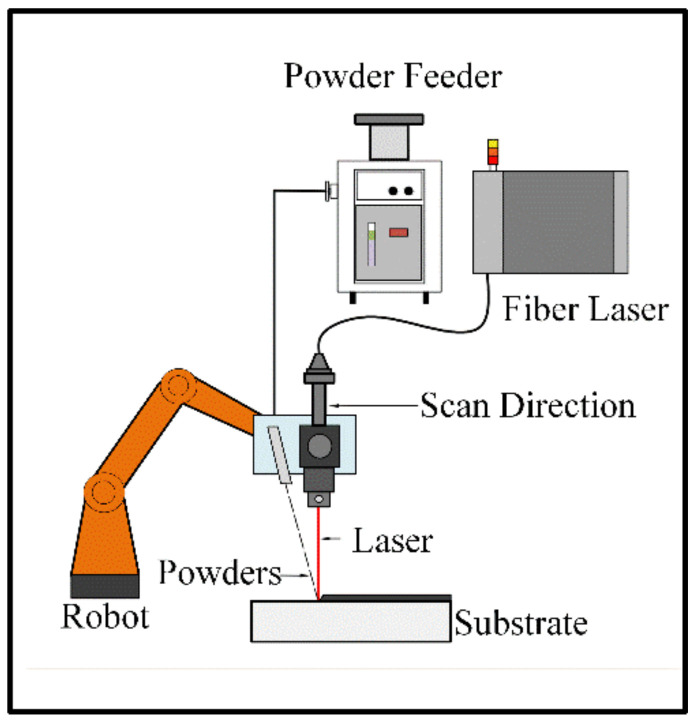
Equipment for the laser melt injection.

As shown in Figure 2, the LMI apparatus included a 6 kW continuous-wave fiber laser (IPG, YLR-6000, IPG Photonics, Oxford, MA, USA) with a laser wavelength of 1.06 μm, a homemade laser head, a 6-axis robot, and a powder feeder (HUST-III, Huazhong University of Science and Technology, Wuhan, China).

**Figure 3:**
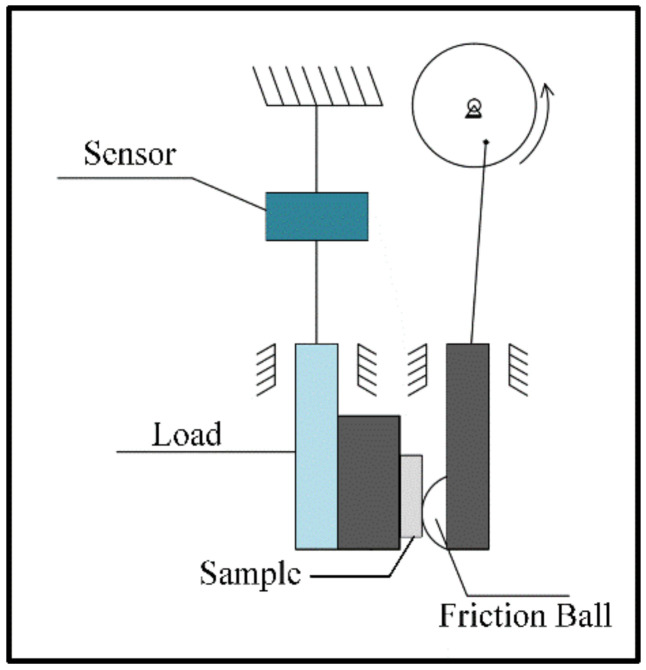
Equipment for the wear test.

During the LMI process, a laser beam with a diameter of 3 mm irradiated the Al substrate’s surface, the W particles were injected into the tail of the laser molten pool, and argon with a flow rate of 4 L/min was used as the delivering and shielding gas. As the laser head scanned, the W particles were captured by the molten pool, and a W/Al composite layer was finally formed, with an overlapping ratio of 50%. In order to investigate the effect of powder feeding rate on laser melt injection, the powder feeding rate was set to 7 g/min, 10 g/min, 13 g/min, or 16 g/min, and the laser power was 3000 W, while the laser scan speed was 700 mm/min. The specimens were machined using an electric spark CNC machine (DK7750, Taizhou Zhongxing CNC Machine Tool Plant, Taizhou, China), and transverse sections of the samples were ground, polished, and then etched with Keller’s reagent for 2–3 s at room temperature. The microstructure of the W/Al composite layer was characterized by scanning electron microscopy (SEM, JSM7600F, Shanghai Baihe instrument Technology Co., Ltd., Shanghai, China) and energy-dispersive spectroscopy (EDS, IncaXMax50, Oxford instruments co., Ltd., Oxford, UK). The chemical composition and elemental distribution of the W/Al composite layer were analyzed with an electronic probe microanalyzer (EPMA, EPMA-8050G, Shimadzu Corporation of Japan, Shimadzu, Japan) equipped with a wavelength-dispersion spectrum (WDS). The phases in the W/Al composite layer were identified with an X-ray diffraction meter (XRD). The hardness of W/Al composite layer was tested using a microhardness tester (HXD-1000TM, Shanghai changfang optical instrument co., ltd., Shanghai, China) with a t load of 2.94 N and holding time of 20 s. The hardness distribution was measured along its depth direction. Room-temperature sliding wear was tested using an abrasion tester (UMT TriboLab, Brooke Technology Co., Ltd., Billerica, MA, USA), as shown in Figure 3, where a Si_3_N_4_ ball with a diameter of 6.3 mm was slid on the specimen with a test load of 15 N and speed of 10 mm/s, the wear length was 5 mm, and the test duration was 20 min. Each group of tests was repeated three times, and the micromorphology of the wear samples was characterized using a confocal microscope (KEYENCE, VK-X2500, Keens Japan Ltd., Osaka, Japan).

## 3. Results

### 3.1. Microstructure

**Figure 4:**
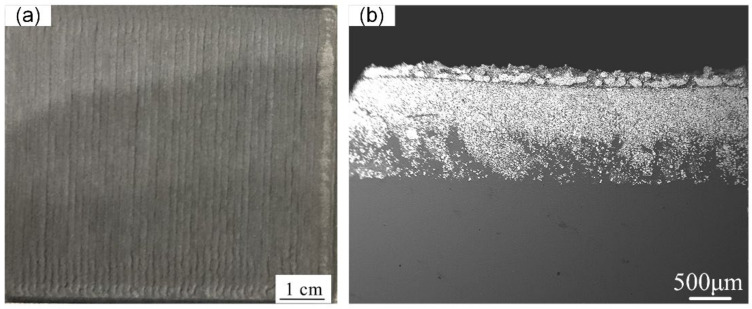
Macroscopic surface (**a**) and cross-section (**b**) of the W/Al composite layer with a powder feeding rate of 16 g/min.

**Figure 5:**
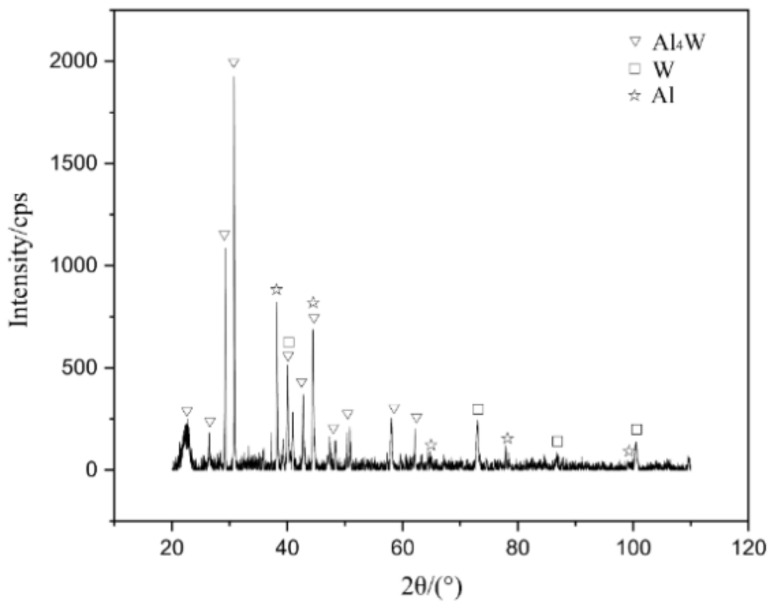
X-ray diffraction spectrum of the W/Al composite layer.

**Figure 6:**
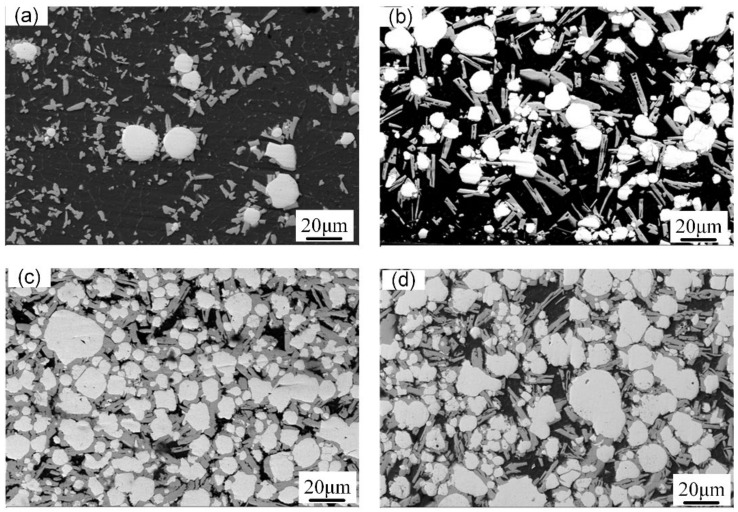
Microstructure of W/Al composite layers with powder feeding rates of (**a**) 7 g/min, (**b**) 10 g/min, (**c**) 13 g/min, and (**d**) 16 g/min.

**Figure 7:**
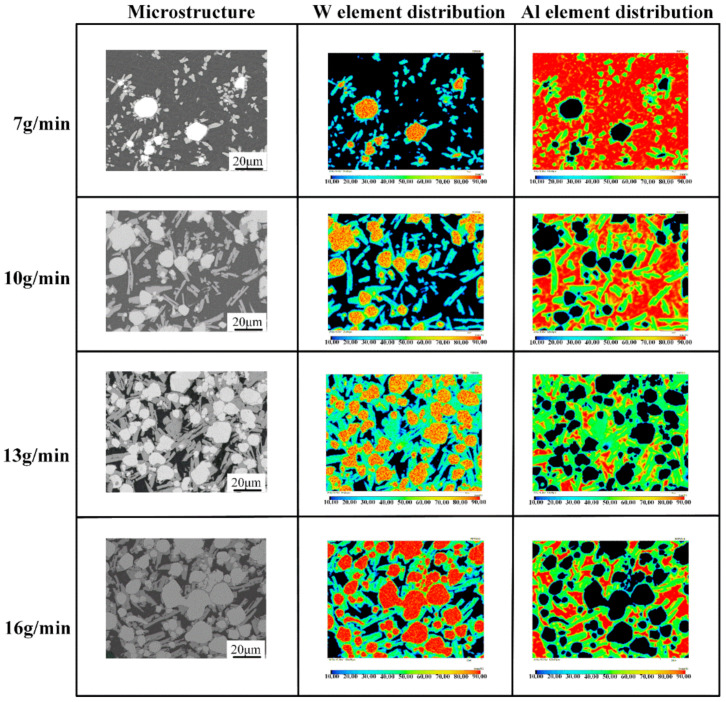
Microstructure and elemental distribution of the W/Al composite layers with powder feeding rates of 7 g/min, 10 g/min, 13 g/min, and 16 g/min.

**Figure 8:**
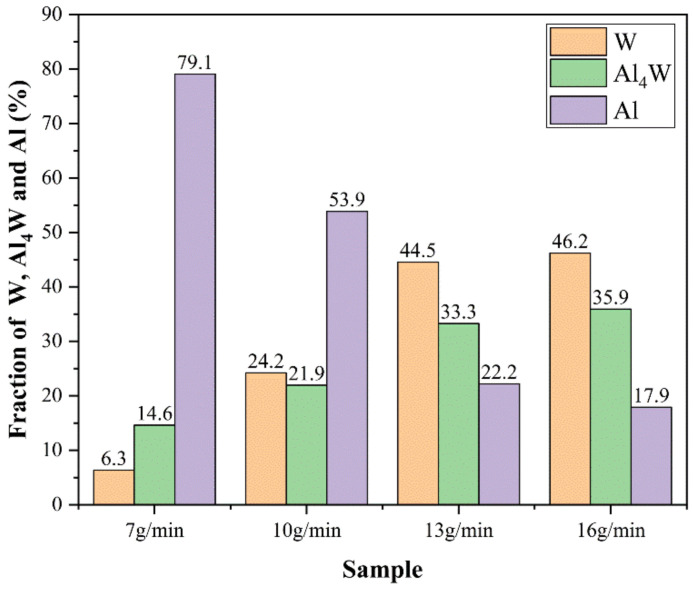
Area fraction of W, Al_4_W, and Al alloy in the W/Al composite layer.

Figure 4 shows the macroscopic surface and cross-section of the W/Al composite layer with a powder feeding rate of 16 g/min. The XRD results in Figure 5 show that W, Al, and Al_4_W are the constituent phases in the W/Al composite layer. The peaks from the aluminum alloy phases are very weak, and the main peak from aluminum is strong in the W/Al composite layer. Figure 6 shows the scanning electron microscopy (SEM) images of the W/Al composite layer. As shown in Figure 6, all of the W/Al composite layers were composed of white particles, dark blocks, and a black matrix. The EPMA results of the W/Al composite layers with powder feeding rates of 7 g/min, 10 g/min, 13 g/min, and 16 g/min are given in Figure 7. The EPMA results show that the composition of the white particles was 100W (at. %), that of the dark block was 79.3Al-20.7W (at. %), and that of the black matrix was 96.5Al-1.3Mg-1.7Zn-0.5Cu (at. %). Based on the EPMA and XRD results, it can be concluded that the white particles are W, the dark blocks are Al_4_W, and the black matrix represents aluminum alloys. The fraction of the reinforcing phase is an important factor that affects the performance of the composites layer. Here, the area fraction of W and Al_4_W in the W/Al composite layer was measured using Imaging-plus 6.0 software (Pro Plus 6.0, American Media Cybernetics image technology company, Rockville, MD, USA), as shown in Figure 8, and the area fraction of W and Al_4_W increased with the increase in the powder feeding rate. When the powder feeding rate was 7 g/min, the area fraction of W particles and Al_4_W in the W/Al composite layer was only 6.3% and 14.6%, respectively. As the powder feeding rate increased to 16 g/min, the area fraction of W particles and Al_4_W increased to 46.2% and 35.9%, respectively.

### 3.2. Hardness

**Figure 9:**
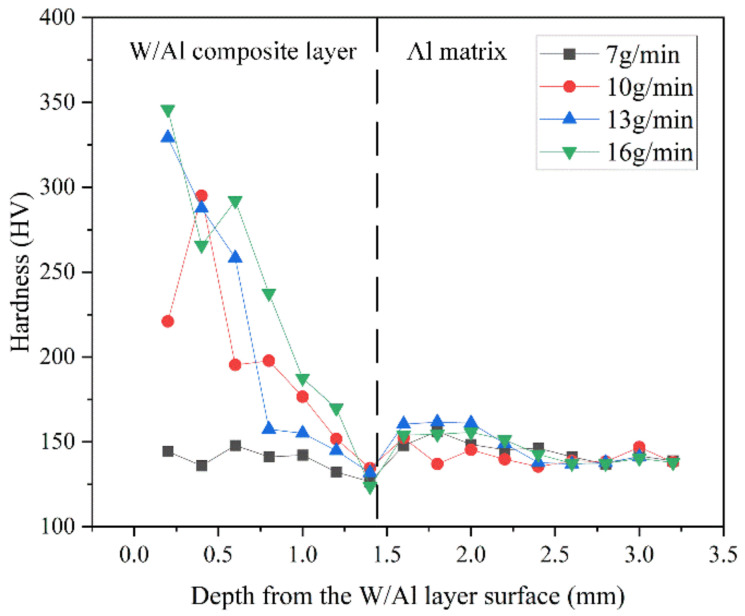
Hardness distribution of the W/Al composite layer.

As shown in Figure 9, the hardness of the W/Al layer increased with the increase in the powder feeding rate. When the powder feeding rate was 7 g/min, the hardness of the W/Al composite layer was almost the same as that of the 7075 Al substrate. As the powder feeding rate increased to 16 g/min, the hardness of the W/Al composite layer could reach up to 350 HV, which is 2.5 times higher than that of the 7075 Al substrate (142.3 HV).

### 3.3. Wear

**Figure 10:**
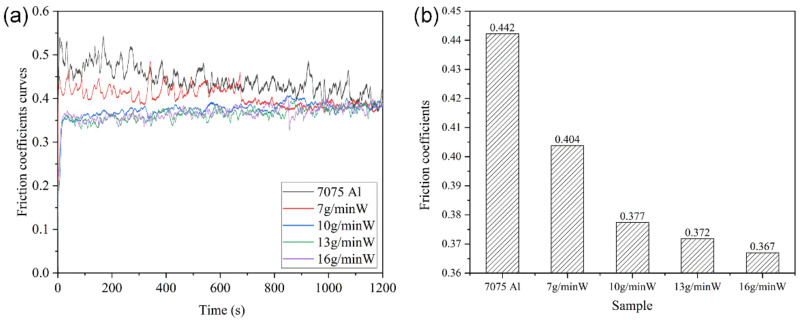
(**a**) Friction coefficient curves and (**b**) average friction coefficients of the W/Al composite layers and 7075 Al substrate.

As shown in Figure 10, all of the W/Al composite layers exhibited lower friction coefficients than that of the 7075 Al substrate. The average friction coefficient of the 7075 Al alloy substrate was about 0.442; with the increase in the W powder feeding rate from 7 g/min to 16 g/min, the average friction coefficient decreased from 0.404 to 0.367.

**Figure 11:**
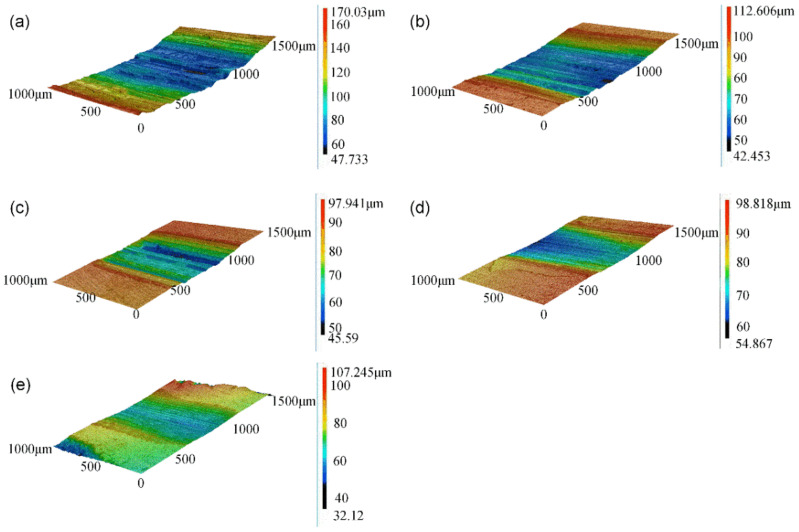
Wear surface morphology of (**a**) 7075 Al alloy and W/Al composite layers with W powder feeding rates of (**b**) 7 g/min, (**c**) 10 g/min, (**d**) 13 g/min, and (**e**) 16 g/min.

**Figure 12:**
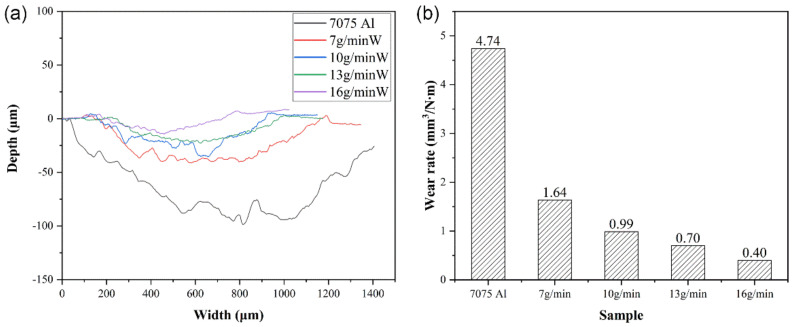
(**a**) Wear scar distribution curves and (**b**) wear rates of 7075 Al alloy and W/Al composite layers.

Figure 11 shows the wear surface of all of the samples; as shown in Figure 11a, the abrasion width and depth of the 7075 Al substrate were 1368.4 μm and 114.7 μm, respectively, while all of the W/Al composite layers exhibited lower abrasion width and depth than those of the 7075 Al substrate. With the increase in the W powder feeding rate from 7 g/min to 16 g/min, the average abrasion width decreased from 1102.2 μm to 617.3 μm, and the average abrasion depth decreased from 50.7 μm to 20.7 μm. According to wear rate, ε = V/(G∙L). The wear volume is V, the test load is G, and the wear scar length is L. As shown in Figure 12, all of the W/Al composite layers exhibited better wear resistance than that of the 7075 Al substrate. The wear rate of the 7075 Al alloy substrate was 4.74 mm^3^/N m; with the increase in the W powder feeding rate from 7 g/min to 16 g/min, the wear rate decreased from 1.64 mm^3^/N m to 0.40 mm^3^/N m.

## 4. Discussion

During the laser melt injection, the W particles enter the high-temperature melt pool, and W atoms diffuse into the laser melt pool. According to the Al–W binary phase diagram, during the cooling process of the melt pool, W particles react with Al solution to form intermetallic compounds. Khoshhal, Niu, and Wang pointed out that Al_4_W was first formed due to its low generation enthalpy, and further calculations show that the low generation enthalpy can be attributed to the fact that Al_4_W has a smaller n(Ef) (the Fermi level) than Al_12_W [18,19,20]. According to the solid–liquid reaction mechanism, Al_4_W is formed by a peritectic reaction between W and aluminum at 1327 °C. At the beginning of the laser melting, the liquid will quickly adhere to the surface of the W, forming an adherent layer with a certain concentration gradient, and the concentration of aluminum gradually decreases from the outside to the inside [21,22]. As the Al concentration in the inside layer of the diffusion layer increases, the solute atom W reacts with the solvent atom Al to form an Al_4_W intermetallic compound enveloping the W particles [23]. There are also partially escaped W and Al reactions to form free Al_4_W intermetallic compounds in the matrix between the W particles. During the process of LMI, the cooling rate is very fast (about 2.8 × 10^3^ °C/s), and there is not enough time for the Al_4_W to react with the molten Al and form Al_5_W and Al_12_W [24]. Therefore, the W/Al composite layer consists of W, Al, and Al_4_W.

**Figure 13:**
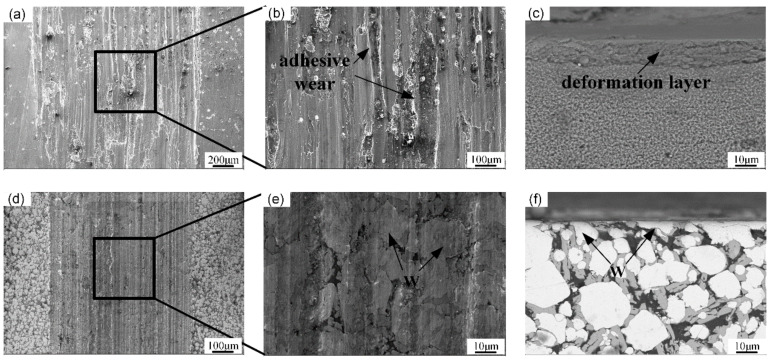
Wear surface (**a**,**b**,**d**,**e**) and cross-sectional view (**c**,**f**) of 7075 Al alloys (**a**–**c**) and W/Al layer with a powder feeding rate of 16 g/min (**d**–**f**).

During the process of wear testing, the temperature of the wear sample is raised due to the friction heat. The 7075 Al alloy has low hardness, and strengthens at elevated temperature; when temperature of the Al alloy reaches its flashpoint, the 7075 Al alloy is welded with the Si_3_N_4_ ceramic ball, and tears under the action of shear force (Figure 13a–c). Thus, the friction coefficient and wear rate of 7075 Al are larger. Compared with the 7075 Al alloy, the W and Al_4_W in the W/Al composite layer have higher hardness at elevated temperature, which can enable them to effectively resist the extrusion of the Si_3_N_4_ ceramic ball, and reduces the wear (Figure 13d–f). Compared with the 7075 Al substrate, the friction coefficient and wear rate of the W/Al composite layer are smaller. This shows that the W/Al composite layer has excellent wear resistance [25].

## 5. Conclusions

(1) A W-particle-reinforced Al matrix composite layer without visible metallurgic defects was prepared via laser melt injection on a 7075 Al alloy substrate, and the composite layer consisted of W, Al_4_W, and Al alloy. The reinforcing phases W and Al_4_W endowed the composite layer with high hardness and excellent wear resistance.
(2) The SEM results show that the W and Al_4_W contents in the W/Al composite layer increased with the increase in the W powder feeding rate; the contents of W particles and Al_4_W were the highest in the W/Al composite layer with a W powder feeding rate of 16 g/min.
(3) With the increase in the W powder feeding rate, the hardness and wear resistance of the W/Al composite layer increased, and the friction coefficient decreased. The hardness of the W/Al composite layer was 1.5–2.5 times higher than that of the 7075 Al. The friction coefficient of the W/Al composite layer was 8.6–17% smaller than that of the 7075 Al, and the wear resistance of the W/Al composite layer was 2.9–11.8 times higher than that of the 7075 Al.

## Figures and Tables

**Table 1:** Chemical composition of the 7075 Al alloy (wt.%).

Elements	Si	Cu	Mg	Zn	Mn	Ti	Cr	Fe	Al
Wt.%	0.40	1.2–2.0	2.1–2.9	5.1–6.1	0.30	0.20	0.18–0.28	0.50	Bal.
